# Waist Circumference-Years Construct Analysis and the Incidence of Type 2 Diabetes: China Health and Nutrition Survey, 1997–2015

**DOI:** 10.3390/nu14214654

**Published:** 2022-11-03

**Authors:** Lijing Xi, Xueke Yang, Ruizhe Wang, Chaoyue Ku, Binbin Wu, Man Dai, Li Liu, Zhiguang Ping

**Affiliations:** 1 College of Public Health, Zhengzhou University, Zhengzhou 450001, China; 2 School of Basic Medical Sciences, Zhengzhou University, Zhengzhou 450001, China

**Keywords:** abdominal obesity, CHNS, type 2 diabetes, waist circumference-years

## Abstract

Background: Few studies have combined the degree and duration of abdominal obesity into a waist circumference-years construct for analysis. The purpose of this study was to investigate the effect of waist circumference-years on the incidence of type 2 diabetes. Methods: A total of 6616 adults from the China Health and Nutrition Survey (CHNS) were enrolled in this study from 1997. The waist circumference-years construct was represented as the sum of the upper and lower area between the waist circumference baseline (men: ≥90 cm, women: ≥85 cm) and the waist circumference line. The correlations in the study were analyzed using logistic regression. Results: The incidence of type 2 diabetes increased with increasing waist circumference-years, with an adjusted risk increase of 38% (95% CI: 31–47%) for each additional 50 waist circumference-years, and this rate was similar across gender and age groups. The area under the curve of waist circumference-years (0.743) was greatest in the receiver operating characteristic curve (ROC) analysis compared to baseline waist circumference (0.731) and the waist-height ratio (0.728) (*p* < 0.05). Conclusion: The waist circumference-years construct is closely associated with an increased risk of type 2 diabetes and may be a stronger predictor of type 2 diabetes risk than baseline waist circumference or the waist-height ratio.

## 1. Introduction

More than 500 million people worldwide already have type 2 diabetes [1], and it is expected to rise to 700 million by 2045 [2]. If the current epidemic is not controlled, diabetes will result in more than 5 million deaths, $1 trillion in healthcare spending, and a huge global economic burden [3]. Moreover, abdominal obesity was an established risk factor for type 2 diabetes [4,5,6]. Waist circumference was established as a reliable and efficient anthropometric measure used as an alternative to abdominal obesity in epidemiological studies [7]. To date, most analyses of abdominal obesity have used waist circumference, the waist-height ratio, or the duration of abdominal obesity as factors in the analysis of the relationship between type 2 diabetes and the risk of related chronic diseases [8,9,10,11], but few studies have combined the degree and duration of abdominal obesity to determine the level of cumulative exposure to abdominal obesity.

Cumulative exposure to abdominal obesity is calculated using the concept of waist circumference-years, which is calculated by adding up the number of waist circumferences above the standard each year. The excessive waist circumference-years construct has been shown to be a predictor of cardiovascular disease risk and may be a stronger predictor than waist circumference alone [12]. The existing studies have only confirmed the association between waist circumference-years and the risk of cardiovascular disease. However, no research has demonstrated the waist circumference-years model’s association with the incidence of type 2 diabetes. Therefore, the objectives of this study were: (1) to explore the association between the waist circumference-years construct and the risk of type 2 diabetes in all participants and in participants stratified by sex and age using the CHNS; and (2) to compare the waist circumference-years model with models of baseline waist circumference or the waist-height ratio alone to determine whether the ability of the waist circumference-years construct to predict type 2 diabetes is superior to that of waist circumference and the waist-height ratio indicators.

## 2. Materials and Methods

### 2.1. Study Participants

CHNS is a cohort study jointly conducted by the University of North Carolina and the Chinese Center for Disease Control and Prevention to explore the dynamics of the Chinese population in terms of social factors such as demographics, economy, and health service levels, so as to offer a basis for relevant policy formulation and adjustment. To date, the project has conducted 10 large surveys in 15 provinces in succession since 1989, covering a wide range of fields including demography, nutrition, health, economics, and sociology [13]. The study was conducted according to the guidelines of the Declaration of Helsinki and approved by the Institutional Review Committees of the University of North Carolina at Chapel Hill and the National Institute of Nutrition and Food Safety, and the Chinese Center for Disease Control and Prevention. All participants in this study provided informed consent.

Due to the absence of important variables in the three surveys in 1989, 1991, and 1993, longitudinal data (*n* = 15,822) from 1997 to 2015 were used in our study, covering a time span of 18 years. A total of 6616 adults were included after the exclusion of minors under 18 years of age (*n* = 5172), participants with missing baseline waist circumference data (*n* = 2216), participants with type 2 diabetes or unknown diagnosis at baseline (*n* = 348), and participants with only baseline information (*n* = 1466).

### 2.2. Variable Measurement

A physical examination was performed by a physician, nurse, health worker, or other professional using standardized methods. Participants were measured for waist circumference and height while wearing light clothing and no shoes, and maintaining a standing posture. Waist circumference was measured as the circumference of a circle made with an inelastic ruler at the midpoint of the lower edge of the lowest rib and the upper edge of the ilium, accurate to 0.1 cm. A portable range finder was used to measure height, accurate to 0.1 cm [14]. Waist-height ratio = waist circumference/height.

This study included basic demographic information such as age, gender, region, and some health-related behavioral factors. Age referred to the time from the date of birth to the time of examination and was divided into two age groups (<60, ≥60). The place of residence was divided into two categories (urban, rural). Marital status (single, married, and widowed, divorced, or separated) and educational attainment (elementary school and below, middle and high school, and college and above) were divided into three categories. Based on self-reported health behaviors, the study categorized participants’ smoking status as never having smoked and smoker; and alcohol consumption as never, no more than 1 time per month, 1–2 times per month, 1–2 times per week, and 3 or more times per week.

### 2.3. The Measurement of Waist Circumference–Years

**Figure 1:**
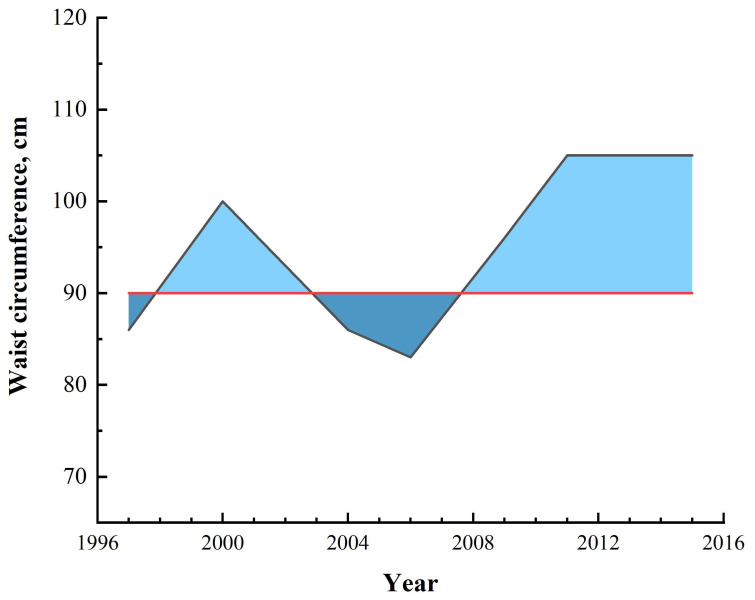
Illustration of waist circumference-year calculation.

To better reflect the change in waist circumference over time, this study used the sum of the upper and lower area between the waist circumference line and the waist circumference baseline (men: ≥90 cm, women: ≥85 cm) [15] to represent the specific value of waist circumference-years (above the baseline was a positive area, below was a negative area. The sum of the two areas was the waist circumference-years. If the waist circumference-years was negative, it was denoted as zero waist circumference-years). For example, seven measurements of the waist circumference of a male subject were 86 cm, 100 cm, 86 cm, 83 cm, 96 cm, 105 cm, and 105 cm, respectively, and the waist circumference-years calculated by trapezoidal rule [16] was 89.5 (Figure 1). As another example, when the calculation results in 50 waist circumference-years, it may be the result of a female waist circumference value of 95 cm lasting five years or a male waist circumference value of 95 cm lasting ten years, and so on in different cases.

### 2.4. Measurement of the Outcome and Time to Event

In this study, type 2 diabetes was determined based on patient self-reporting, fasting glucose ≥7.0 mmol/L, or current treatment with glucose-lowering drugs or insulin. The endpoint of the study was the development of type 2 diabetes, and for those who were lost to follow-up, died, or did not have confirmed type 2 diabetes at the end of the study, the waist circumference-years construct was calculated continuously until the period of death, or the date of the last follow-up visit performed.

### 2.5. Statistical Analysis

The demographic and health behavior characteristics of participants were described as mean ± standard deviation for continuous variables and *n* (%) for categorical variables. The *T*-test and chi-square test were used for the comparison of the mean and the prevalence between subjects with and without diabetes. The incidence of type 2 diabetes per 1000 person-years in the study was calculated as the degree of the accumulation of waist circumference-years. The difference between the time to diagnosis of type 2 diabetes and the baseline was the risk follow-up time. For participants without type 2 diabetes, follow-up was based on the time of death or the last survey.

Logistic regression analysis was used in this study to estimate the odds ratios (ORs) and 95% confidence intervals (CIs). When analyzed as a continuous variable, the waist circumference-year was described by each 50-unit increase in waist circumference-years for comparison with the existing literature [12]; when used as a categorical variable, it was divided into five groups (0, 1–49.9, 50–99.9, 100–149.9, and ≥150). The study was analyzed for all participants and the category of waist circumference-years was stratified by sex and age. The variables adjusted for use in the analysis of model 1 were baseline age and gender. Model 2 adjusted variables for baseline age, gender, residence, marital status, and education level. Model 3, on the other hand, adjusted for two additional health behavior factors, smoking and alcohol consumption, in addition to model 2. The area under the ROC curve (AUC) was obtained from the ROC to compare the ability of the waist circumference-years model to predict type 2 diabetes with a model that included only baseline waist circumference or the waist-height ratio. Analyses were performed using IBM SPSS Statistics 26.0 software (IBM Corp., Armonk, NY, USA) and MedCalc 20.1.0 statistical software (MedCalc Software Ltd, Ostend, Belgium) with a test level of α = 0.05.

## 3. Results

### 3.1. Characteristics of the Participants

6616 participants did not have type 2 diabetes in the baseline study and 315 had type 2 diabetes after a median follow-up of 14 (3–18) years. The mean age of the participants at baseline was 43.4 years and 51% were female. The differences in age, baseline waist circumference, waist circumference-years, residence, and marital status in the type 2 diabetes group were statistically significant compared with the non-diabetic group (*p* < 0.001) (Table 1).

### 3.2. Incidence Rate and Odds Ratios of Type 2 Diabetes

In a follow-up survey of 87,457 person-years, the result of the sum of the positive and negative area of waist circumference-years was less than the standard area in 76.3% of participants, recorded as zero waist circumference-years, who were classified as non-abdominal obese. With the increasing waist circumference-years, the incidence of type 2 diabetes showed a corresponding increasing trend. The incidence of type 2 diabetes in waist circumference-years grouped 0, 1–49.9, 50–99.9, 100–149.9, and ≥150 was 2.05, 5.54, 7.98, 9.45, and 13.42 per 1000 person-years, respectively. The corrected ORs (model 3) for type 2 diabetes in the four subgroups, 1–49.9, 50–99.9, 100–149.9, and ≥150 waist circumference-years, compared to zero waist circumference-years were 2.63 (95% CI: 1.80–3.83), 3.56 (95% CI: 2.43–5.20), 4.39 (95% CI: 2.94–6.56), and 5.75 (95% CI: 4.14–8.00), respectively (*p*-trend < 0.001). In addition, when the study used continuous variables, the adjusted OR for type 2 diabetes was 1.38 (95% CI: 1.31–1.47) for every 50 waist circumference-years increase (Table 2).

### 3.3. Subgroup Analysis Results

The study was grouped overall by gender and age to compare the correlation between waist circumference-years and type 2 diabetes across gender and age groups. In the gender subgroup, the adjusted ORs for type 2 diabetes per 50 waist circumference-years increase was 1.35 (95% CI: 1.23–1.48) for men and 1.41 (95% CI: 1.30–1.52) for women. This meant that this rate was 6% higher for women than for men. In the age subgroups, the corrected ORs for type 2 diabetes were 1.42 (95% CI: 1.33–1.52) and 1.24 (95% CI: 1.10–1.40) for each additional 50 waist circumference-years in the subgroups <60 years and ≥60 years, respectively. This rate also varied by age subgroup and was higher for those under 60 years than for those 60 years and older (Table 3).

### 3.4. ROC Comparison of Different Indexes

To compare the ability of the commonly used baseline waist circumference, the waist-height ratio, and waist circumference-years to predict type 2 diabetes, this study used ROC for analysis. The AUC for waist circumference-years was 0.743 (95% CI: 0.715–0.770), which was greater than baseline waist circumference [0.731 (95% CI: 0.703–0.759)] and the waist-height ratio [0.728 (95% CI: 0.700–0.757)], and the sensitivity was higher when waist circumference-years was used for prediction. All comparisons between groups were statistically different (*p* < 0.05). It can be concluded that waist circumference-years was superior to baseline waist circumference and the waist-height ratio in terms of their ability to predict type 2 diabetes. Furthermore, similar outcomes existed in both gender and age subgroups (Table 4).

## 4. Discussion

Using an 18-year follow-up survey of adult Chinese residents, the same trend was observed for the incidence of type 2 diabetes as waist circumference-years increased. Waist circumference-years was better able to predict the risk of type 2 diabetes compared to baseline waist circumference and waist-height ratio models. These results suggest that, in the future, higher exposure to and the longer duration of abdominal obesity will adversely affect the prevention of type 2 diabetes, which will further increase the burden of disease associated with abdominal obesity.

Existing studies on the relationship between obesity-years (cumulative exposure to the degree and duration of obesity) and the risk of disease have yielded similar results to this study. The results from a longitudinal study of reproductive health in Australian women aged 18–23 years, starting in 1996, showed a positive association between the occurrence of type 2 diabetes and the years of obesity, degree of obesity, and obesity-years (*p*-trend < 0.001) [17]. The Framingham Heart Study found an adjusted hazard ratio for type 2 diabetes of 1.07 (95% CI: 1.06–1.09) for every 10 obesity-years increase after 48 years of follow-up in 5036 participants [18]. In addition, results from some studies suggest that waist circumference performs better than BMI (body mass index) in predicting outcomes related to metabolically related diseases, which may also mean that waist circumference-years is a better predictor than obesity-years [7,19,20]. Based on these studies, we were eager to understand whether there was such an association between waist circumference-years and abdominal obesity-related diseases. One of the studies using the development of coronary risk in youth confirmed the association of waist circumference-years with the risk of cardiovascular disease and the ability to predict this disease [12]. These findings may be due to the fact that the persistence of obesity, especially abdominal obesity, can adversely affect the body’s metabolism, which in turn can lead to the development of disease [21,22]. Therefore, the present study used waist circumference-years as a new predictive model, confirming its predictability for type 2 diabetes, an indicator that could be applied to other obesity-related diseases in the future.

In a subgroup analysis of sex, we found that the risk of type 2 diabetes varied by sex with increasing waist circumference-years, and was higher in women than in men. This finding was echoed in similar studies. For example, a UK study showed that the adjusted hazard ratio for type 2 diabetes was 3.53 (95% CI: 1.92–6.48) for the highest compared to the lowest quartile of waist circumference in men and the corresponding adjusted relative risk for women was 12.18 (95% CI: 4.83–30.74) in a 20-year follow-up survey [23]. This difference may be explained by the higher skeletal muscle mass in men than in women [24], a factor that may promote insulin resistance [25]. In addition, the effects of sex chromosomes and sex-specific hormones such as estrogen further contribute to this difference [26].

Similarly, age had an effect on the association between waist circumference-years, the indicator in the study, and type 2 diabetes. There may be several reasons why the risk of type 2 diabetes is higher in people younger than 60 than in people 60 and older. First, as we age, the body composition of the human body changes at different stages, either in the form of changes in whole-body muscle mass or fat, or more specifically, in the form of changes in individual body weight. As a result, waist circumference measures may not quantify body fat well in older adults [27,28]. Second, the strength of the association between waist circumference and visceral fat is inconsistent across studies due to differences in age, gender, race, and health status [29]. In addition, the duration and survival of type 2 diabetes may differ significantly for older and young adult groups, and for older adults, moderate obesity may have a protective effect on health, further contributing to differences in findings across ages [30,31,32]. Moreover, it is worth noting that people who were obese in adolescence had a higher risk of developing diabetes compared to those who became obese in adulthood [33]. This may be partly due to the greater degree of insulin resistance to obesity in younger compared to older age groups with abdominal obesity [34,35], and could be partly related to the fact that obesity also lasts longer in people who become obese earlier, both of which may be risk factors for poor health status in younger age groups [8,36]. Therefore, the prevention of type 2 diabetes should focus more on the young group, try to control body weight, delay the occurrence of obesity, and reduce the time of obesity.

Among the existing studies comparing the ability of different baseline obesity metrics to predict type 2 diabetes, it remains controversial as to which metric has better predictive power [9,37,38,39,40]. One of these studies found that indicators of abdominal obesity performed better than general indicators of overweight or obesity in predicting type 2 diabetes [9,41]. However, the results of the previous study only proved that the AUC of waist circumference and the waist-height ratio, indicators of abdominal obesity, were greater than BMI, and did not prove that there was a difference between these two indicators of abdominal obesity, which were considered to have the same predictive power [9]. In this study, we found that waist circumference differed from the waist-height ratio in its ability to predict type 2 diabetes and that waist circumference was greater than the waist-height ratio. Most notably, we found that the AUC of the waist circumference-years indicator was significantly greater than both baseline abdominal obesity indicators. This suggests that waist circumference-years was a better predictor of the risk of type 2 diabetes in this study than an indicator that includes only baseline abdominal obesity.

This study has several strengths. (1) The CHNS survey used in this study has a reasonable design, covers a wide range of people, involves a large number of people, has comprehensive survey factors, and has a long follow-up period, which is very representative and practical. (2) The waist circumference-years construct was specifically calculated to better reflect the dynamic changes in the waist circumference values of participants during follow-up. (3) The study compared the relationship between waist circumference-years and the risk of type 2 diabetes by sex and age, and further analyzed the ability of waist circumference-years to predict type 2 diabetes with abdominal obesity indicators in each subgroup. However, there are several shortcomings in this study. (1) Type 2 diabetes was associated with genetic factors, but CHNS lacked investigations on the genetic aspects of diabetes, and a portion of the identification of type 2 diabetes in the CHNS database was based on self-reporting, which may underestimate the outcome events to some extent. (2) The study adjusted for major confounders, but there are still some confounders that were not adjusted for, such as diet, etc. (3) Waist circumference, as a simple estimator of abdominal obesity, does not provide an accurate estimate of abdominal fat mass. The quantitative instruments for abdominal obesity also have various limitations in terms of cost, technology, and space [42]. Therefore, in future studies, it may be better to use metrics such as abdominal fat mass percentage (AFM%) to accurately estimate abdominal obesity [43].

## 5. Conclusions

Waist circumference-years was a joint indicator of the extent and duration of abdominal obesity. Furthermore, the waist circumference-years construct was better than baseline waist circumference or the waist-height ratio alone in predicting the risk of type 2 diabetes in all cases in this study. These results suggest that for the future prevention of type 2 diabetes, the focus should be on avoiding or delaying the onset of obesity in younger age groups and on reducing the time spent in obesity for those who are already obese.

## Figures and Tables

**Table 1:** Participant characteristics of the diabetic and non-diabetic study populations ^a^.

Variable	Overall (*n* = 6616)	Diabetes (*n* = 315)	No Diabetes (*n* = 6301)	*p*-Value
Age (years)	43.4 ± 14.6	50.2 ± 11.4	43.0 ± 14.7	<0.001
Waist circumference (cm)	77.4 ± 9.2	85.3 ± 9.9	77.3 ± 9.0	<0.001
Waist circumference-years	25.1 ± 0.8	76.3 ± 5.8	22.5 ± 0.7	<0.001
**Sex**				0.337
Men, *n* (%)	3241 (49.0)	146 (46.3)	3095 (49.1)	
Women, *n* (%)	3375 (51.0)	169 (53.7)	3206 (50.9)	
**Area**				<0.001
Urban, *n* (%)	2233 (33.8)	143 (45.4)	2090 (33.2)	
Rural, *n* (%)	4383 (66.2)	172 (54.6)	4211 (66.8)	
**Smoking**				0.408
Never, *n* (%)	4416 (66.7)	217 (68.9)	4199 (66.6)	
Smoker, *n* (%)	2200 (33.3)	98 (31.1)	2102 (33.4)	
**Alcohol drinking**				0.056
never	4212 (63.7)	202 (64.1)	4009 (63.6)	
no more than 1 time per month	700 (10.6)	44 (14.0)	656 (10.4)	
1–2 times per month	390 (5.9)	24 (7.6)	366 (5.8)	
1–2 times per week	585 (8.8)	22 (7.0)	563 (8.9)	
3 or more times per week	728 (11.0)	23 (7.3)	446 (11.2)	
**Marital status**				<0.001
Single	683 (10.4)	9 (2.9)	674 (10.8)	
Married	5491 (83.6)	284 (90.7)	5207 (83.2)	
Widowed, divorced or separated	396 (6.0)	20 (6.4)	376 (6.0)	
**Educational**				0.231
Primary school or lower	3563 (54.4)	184 (59.0)	3379 (54.2)	
Junior or Senior Secondary	2867 (43.8)	122 (39.1)	2745 (44.0)	
Junior college or above	116 (1.8)	6 (1.9)	110 (1.8)	

^a^ Values are expressed as mean ± SD for continuous variables or *n* (%) for categorical variables.

**Table 2:** The incidence, odds ratio, and 95% confidence interval for type 2 diabetes according to waist circumference-years.

	Waist Circumference-Years (cm·Years)						Per 50 Waist Circumference-Years
	0 (*n* = 5052)	1–49.9 (*n* = 505)	50–99.9 (*n* = 387)	100–149.9 (*n* = 283)	≥150 (*n* = 389)	*p*-Trend	
No. of events	137	39	39	36	64		
No. of person-years	66,945	7043	4888	3811	4770		
Incidence rate ^a^	2.05	5.54	7.98	9.45	13.42		
Model 1 ^b^	1.00 (ref)	2.86 (1.98–4.14)	3.68 (2.52–5.36)	4.83 (3.26–7.16)	6.05 (4.37–8.37)	<0.001	1.40 (1.32–1.48)
Model 2 ^c^	1.00 (ref)	2.64 (1.81–3.84)	3.60 (2.46–5.25)	4.43 (2.97–6.62)	5.78 (4.16–8.02)	<0.001	1.38 (1.31–1.47)
Model 3 ^d^	1.00 (ref)	2.63 (1.80–3.83)	3.56 (2.43–5.20)	4.39 (2.94–6.56)	5.75 (4.14–8.00)	<0.001	1.38 (1.31–1.47)

^a^ Per 1000 person-years. ^b^ Adjusted for age and sex. ^c^ Adjusted for age, sex, area, marital status, and educational level. ^d^ Adjusted for age, sex, area, marital status, educational level, smoking, and alcohol drinking.

**Table 3:** The odds ratio and 95% confidence interval for type 2 diabetes according to waist circumference-years category.

	Model 1 ^a^	Model 2 ^b^	Model 3 ^c^
**Men**			
0	1.00 (ref)	1.00 (ref)	1.00 (ref)
1–49.9	2.61 (1.53–4.45)	2.25 (1.30–3.89)	2.19 (1.26–3.80)
50–99.9	3.34 (1.83–6.08)	2.97 (1.62–5.42)	2.87 (1.56–5.26)
100–149.9	3.78 (1.98–7.22)	3.22 (1.68–6.29)	3.12 (1.62–6.03)
≥150	5.43 (3.28–9.00)	4.75 (2.85–7.90)	4.70 (2.81–7.85)
*p*-trend	<0.001	<0.001	<0.001
Per 50 waist circumference-years	1.39 (1.27–1.52)	1.35 (1.23–1.48)	1.35 (1.23–1.48)
**Women**			
0	1.00 (ref)	1.00 (ref)	1.00 (ref)
1–49.9	3.20 (1.91–5.37)	3.02 (1.78–5.10)	3.04 (1.79–5.15)
50–99.9	4.11 (2.50–6.73)	4.21 (2.55–6.95)	4.17 (2.53–6.89)
100–149.9	5.86 (3.53–9.74)	5.50 (3.26–8.28)	5.49 (3.25–9.29)
≥150	6.82 (4.42–10.54)	6.83 (4.38–10.65)	6.82 (4.38–10.64)
*p*-trend	<0.001	<0.001	<0.001
Per 50 waist circumference-years	1.41 (1.31–1.52)	1.41 (1.30–1.51)	1.41 (1.30–1.52)
**<60**			
0	1.00 (ref)	1.00 (ref)	1.00 (ref)
1–49.9	2.50 (1.63–3.84)	2.42 (1.57–3.72)	2.41 (1.56–3.70)
50–99.9	3.99 (2.59–6.15)	3.88 (2.52–5.98)	3.76 (2.44–5.80)
100–149.9	5.46 (3.54–8.42)	5.16 (3.32–8.02)	5.03 (3.23–7.84)
≥150	6.82 (4.68–9.95)	6.45 (4.41–9.43)	6.36 (4.34–9.32)
*p*-trend	<0.001	<0.001	<0.001
Per 50 waist circumference-years	1.44 (1.35–1.54)	1.43 (1.33–1.52)	1.42 (1.33–1.52)
**≥60**			
0	1.00 (ref)	1.00 (ref)	1.00 (ref)
1–49.9	3.16 (1.48–6.75)	2.51 (1.11–5.66)	2.44 (1.07–5.58)
50–99.9	2.44 (1.11–5.34)	2.37 (1.05–5.31)	2.32 (1.03–5.26)
100–149.9	2.35 (0.87–6.37)	1.99 (0.77–5.50)	1.84 (0.66–5.12)
≥150	3.57 (1.87–6.83)	3.47 (1.77–6.80)	3.41 (1.73–6.71)
*p*-trend	<0.001	<0.001	<0.001
Per 50 waist circumference-years	1.25 (1.12–1.41)	1.25 (1.11–1.41)	1.24 (1.10–1.40)

^a^ Adjusted for age and sex. ^b^ Adjusted for age, sex, area, marital status, and educational level. ^c^ Adjusted for age, sex, area, marital status, educational level, smoking, and alcohol drinking.

**Table 4:** ROC curve analysis results.

Category	AUC	95% CI	*p* Value	Cutoff Value	Sensitivity (%)	Specificity (%)
Waist circumference	0.731	0.703–0.759	<0.001	81.500	0.631	0.715
Waist height ratio	0.728	0.700–0.757	<0.001	0.508	0.666	0.703
Waist circumference-years	0.743	0.715–0.770	<0.001	0	0.720	0.688
**Men**						
Waist circumference	0.725	0.682–0.767	<0.001	84.500	0.596	0.762
Waist height ratio	0.724	0.681–0.768	<0.001	0.508	0.616	0.769
Waist circumference-years	0.733	0.692–0.774	<0.001	0	0.788	0.604
**Women**						
Waist circumference	0.741	0.704–0.779	<0.001	81.500	0.601	0.750
Waist height ratio	0.732	0.695–0.770	<0.001	0.509	0.708	0.648
Waist circumference-years	0.754	0.716–0.791	<0.001	0	0.768	0.659
**<60**						
Waist circumference	0.744	0.713–0.776	<0.001	81.500	0.640	0.735
Waist height ratio	0.742	0.710–0.774	<0.001	0.508	0.657	0.736
Waist circumference-years	0.754	0.723–0.786	<0.001	0	0.715	0.703
**≥60**						
Waist circumference	0.669	0.605–0.733	<0.001	86.333	0.528	0.745
Waist height ratio	0.652	0.587–0.718	<0.001	0.524	0.625	0.633
Waist circumference-years	0.689	0.630–0.748	<0.001	0	0.722	0.636

## Data Availability

Data are available in a public, open access repository: China Health and Nutrition Survey—China Health and Nutrition Survey (CHNS) (unc.edu).
